# Radioimmunotherapy: a brief overview

**DOI:** 10.2349/biij.2.3.e23

**Published:** 2006-07-01

**Authors:** DCE Ng

**Affiliations:** Department of Nuclear Medicine and PET, Singapore General Hospital, Singapore

**Keywords:** Monoclonal antibodies, oncology, ibritumomab tiuxetan, tositumomab

## Abstract

With the advent of biotechnological advances and knowledge of molecular and cellular biology, radioimmunotherapy (RIT) has become a highly promising oncologic therapeutic modality with established clinically efficacy, particularly in non-Hodgkin’s lymphomas. This paper provides a short survey of the basic science of RIT and the various monoclonal antibodies and radionuclides used. A brief review of the published literature on the clinical applications of radioimmunotherapy, particularly in non-Hodgkin’s lymphoma, is provided. New research data indicate many potential areas of development of this modality, including haematological and solid-organ radioimmunotherapy as well as new radionuclidic approaches and clinical protocols.

## INTRODUCTION

Radioimmunotherapy (RIT) evolved from the spectacular growth in molecular biology and biotechnological advances that resulted in the production of highly purified monoclonal antibodies for clinical use. Just as radio-labelling has been very successful with organic ligands such as bisphosphonates for bone scan, or small peptides for octreotide scanning, highly specific and purified monoclonal antibodies are excellent targets for radiochemical labelling for diagnostic and therapeutic purposes. It has been more than two decades since the early reports of radio-labelled antibodies for diagnostic purposes, such as in Tc99m-anti-CEA antibodies, for imaging of metastatic sites of colorectal carcinoma. The current interest is more in the potential of radio-labelled monoclonal antibodies for therapeutic purposes. Highly specific and purified, but non-radioactive, monoclonal antibodies have been used in clinical practice for various medical indications with good results. For instance, ritximab (Mabthera) has been used against the CD20 antigen on B-cell non-Hodgkin’s lymphomas (NHLs), and trastuzumab (herceptin) has been directed at the human epidermal growth factor receptor 2 (HER-2) in breast cancer.

Such highly specific ligands may act as targeted therapeutic agents, delivering adequate radiation dose at specific tumour sites, “guided” by monoclonal antibodies that are clearly antigen-specific. Conversely, this methodology is expected to reduce radiation dose to other tissues, especially critical organs such as the haematopoietic system.

The efficacy of RIT rests on three fundamental principles: cellular biology, monoclonal antibody selection and radionuclide selection. It begins with the identification of appropriate cellular targets favourable to the creation of a potential in-vivo nuclear medicine therapy. Haematological malignancies can exploit this methodology. They typically express various types of antigens on their cell surface, depending on cell type and cellular differentiation. For instance, acute lymphoblastic leukaemia (ALL) expresses CD5, CD22 and CD45, while acute myeloid leukaemia (AML) expresses CD15, CD33. NHLs express various antigenic types such as CD19, CD20, CD21 and CD22. Most experience has been reported in RIT for NHLs directed at the CD20 antigen. For NHLs, the B-cell antigen CD20 is expressed in high density on B-cell malignancies. CD20 is a B-cell antigen present on the surface of normal B cells, pre-B cells, and more than 90% of B-cell lymphomas, but it is not found on B-cell precursors, plasma cells, or other non-lymphoid normal tissues. Upon binding, the antigen-antibody complex is internalised, nor is it shed or secreted. These favourable features allow the radio-labelled antibody to remain on the cell surface to exert its desired therapeutic effects.

The clinical efficacy of RIT is distinct from conventional external beam radiotherapy and systemic chemotherapy in that it involves continuous exposure to low-dose radiation that slowly decreases over time. A study in mice with Burkitt’s lymphoma suggests that it works through cell apoptosis (programmed cell death), rather than by cell necrosis [1].

### Monoclonal antibodies

In modern molecular and cellular biotechnology, monoclonal antibodies can be produced in significant amounts for therapy. Many studies were conducted when only murine antibodies were available for radio-labelling. More recently, chimeric and even humanised monoclonal antibodies have become more widespread in clinical use.

In the literature of RIT involving NHLs, most of the antibodies used were IgGs (Table 1). In fact, several commercially available monoclonal antibodies have been produced and used in various clinical indications (Table 2).

**Table 1:** Antibodies developed for RIT

**Antibody**	**Antigen**	**Type of antibody**
Lym-1	HLA-DR10	murine IgG2a
anti-B1	CD20	murine IgG2a
2B8	CD20	murine IgG1
C2B8	CD20	chimeric IgG1
hLL2	CD22	humanised IgG1
MB-1	CD37	murine IgG1
Campath-1H	CD52	humanised IgG

**Table 2:** Some commercially available monoclonal antibodies and their clinical use

**Monoclonal Antibodies**	**Target**	**Medical Use**
OKT3	CD3 antigens on T-lymphocytes	Acute rejection of transplanted kidneys, hearts and livers
Abciximab	GP IIb/IIIa on platelets	Anti-thrombotic applications
Rituximab	CD20 receptors on B lymphocytes	Non-Hodgkin’s lymphoma
Daclizumab	Interleukin-2 receptors on activated T lymphocytes	Acute rejection of transplanted kidneys
Trastuzumab (Herceptin)	HER-2 growth factor receptors	Advanced breast carcinomas expressing HER-2 receptors
Inflixibmab	TNF (tumour necrosis factor)	Rheumatoid arthritis and Crohn’s disease
Basiliximab	Interleukin-2 receptors on activatedT lymphocytes	Acute rejection of transplanted kidneys
Palivizumab	F protein of respiratory syncytial virus (RSV)	RSV infection in children
Gemtuzumab	CD33 antigen	Relapsed acute myeloid leukemia
Alemtuzumab	CD52 antigen on B and T lymphocytes	B-cell chronic lymphocytic leukaemia
Cetuximab	EGFR (epidermal growth factor receptor)	Colorectal carcinoma and some other tumours

### Radionuclides

The selection of an appropriate radionuclide is crucial in the overall design of a clinically useful RIT. The suitability of a radionuclide resides in its physical and chemical properties; its capacity for conjugation with organic ligands; its stability in-vivo after conjugation; the nature of its radiation; and the clearance behaviour of the isotope-complex. The choice of a radionuclide is also influenced by the clinical disease, such as tumour size, physiological behaviour and tumour radio-sensitivity.

Nuclides with beta radiation (β) are crucial to RIT and produce cellular damage due to the ionising properties of beta radiation. There is the additional effect of cross-fire where surrounding bystander cells, which did not receive enough complex binding, are also destroyed by radiation from adjacent targeted cells. Those beta emitter nuclides, with additional production of gamma radiation (β/γ), allow for dosimetry and imaging. But this additional long-range gamma radiation would usually require the isolation of the patient to reduce radiation exposure to the public.

The two most widely used radionuclides in RIT are Iodine-131 and Yttrium-90 (Table 3). Iodine-131 has a physical half-life of about 8 days (193 hours) and produces gamma radiation for imaging. It is relatively inexpensive and readily available. Yttrium-90 has a higher energy emission and longer path length. It is suitable for irradiation of larger tumours, but the absence of gamma emission prevents its use for imaging.

**Table 3:** Radionuclides used in RIT

**Isotope**	**Half-life (hrs)**	**Radiation**	**Max. energy (keV)**	**Max. range (mm)**
Iodine-131	193	β or γ	610	2.0
Yttrium-90	64	β	2,280	12.0
Rhenium-186	91	β	1,080	5.0
Rhenium-188	17	β or γ	2,120	11.0
Copper-67	62	β	577	1.8
Bismuth-213	77	α	> 6,000	< 0.1
Astatine-211	7	α	7,450	0.1

### Radiochemical conjugation

Different chemical synthetic pathways have been developed for chemically linking radionuclides to monoclonal antibodies. Zevalin links the yttrium nuclide through a specific linker molecule, tiuxetan, to the parent monoclonal antibody (ibritumomab) via thiourea bonds to lysine and arginine amino acids in the Fc portion of the immunoglobulin. Bexxar directly links the iodine nuclide to the antibody via covalent bonds to tyrosine amino acids in the antibody molecule, tositumomab.

### RIT in NHLs

Early papers on RIT for NHLs were on refractory or relapsed NHLs that had failed conventional chemotherapy and radiation therapy. In the late 1980s, DeNardo *et al*, one of the early groups working on RIT, treated 18 patients of B-cell NHL with Iodine-131 conjugated-Lym-1. Since then, many papers on RIT for NHLs have been published involving Phase I/II or II trials. Several papers have also been published in which high dose marrow-ablative RIT has been used in conjunction with bone marrow transplant rescue.

Phase II clinical trials were performed for pre-registration of both the commercial preparations of I-131 tositumomab and Y-90 ibritumomab in patients with indolent lymphoma (follicular), whose disease had become refractory to conventional therapy. The results of these trials were highly promising, with a reported response rate of 70% for I-131 tositumomab and 74% for Y-90 ibritumomab tiuxetan, and a complete response rate of 32% for I-131 tositumomab and 16% for Y-90 ibritumomab tiuxetan. There was median response duration of 15.4 months in I-131 tositumomab and in excess of 7.7 months in Y-90 ibritumomab. The group of patients that received Y-90 ibritumomab tiuxetan had a higher proportion of bulky disease, which may account for the apparent difference in the complete response rate and duration of response between the two therapies.

The main adverse effect reported was marrow suppression, with neutropenia and thrombocytopenia being the most common haematological events. In a small proportion of patients, red cell and platelet transfusions were necessary. There was a small risk of infection requiring hospitalisation. Non-haematological adverse effects, which were generally of minor significance, included nausea, chills, fever, headache and rashes. The development of HAMA (human anti-mouse-antibodies) was low and was reported as 8% for I-131 tositumomab and 1% for Y-90 ibritumomab.

A serious potential concern is the risk of the development of myelodysplasia and/or acute myeloid leukaemia. Although this has been observed in a few treated patients, it is currently not clear whether it is due to the effects of RIT or prior chemotherapy.

Of significance is a recent paper, following up on 1,071 patients who had enrolled in seven studies using I-131 tositumomab for RIT of NHL, which showed that out of 25 confirmed cases of treatment-related myelodysplastic syndromes and acute myeloid leukaemia, 52% developed after RIT with I-131 tositumomab. This represents a crude incidence of 2.3% and an annualised incidence of 1.1% per year, which compares favourably with reported rates following chemotherapy used in the treatment of low-grade NHL. For a small group of patients that received I-131 tositumomab as the initial therapy, the median follow-up approaching five years showed no case of treatment-related myelodysplastic syndromes and acute myeloid leukaemia. These findings are encouraging, although longer follow-up studies are needed [2].

Other than Zevalin and Bexxar, I-131 rituximab RIT has also been developed and results of a Phase II clinical trial has shown high radiochemical purity and preservation of immunoreactivity. In such studies, pre-therapeutic loading of unlabelled rituximab was followed by administration of I-131 rituximab, calculating dosing based on dosimetric studies to deliver a whole body radiation absorbed dose of 75 cGy. Rituximab is a commercially available chimeric IgG1 anti-CD20 monoclonal antibody, with similarities to the murine antibodies used in Bexxar. The objective response rate (ORR) was 71% in 35 patients with median follow-up of 14 months. Complete remission was achieved in 54% of patients with median duration of 20 months [3].

### Schedule of Y-90 ibritumomab tiuxetan therapy and clinical efficacy

The schedule for Y-90 ibritumomab tiuxetan RIT includes several steps. A ‘cold’ therapeutic dose of rituximab (250 mg/m2) is given one week prior to the treatment to optimise tumour targeting. This is to deplete circulating CD20+ B cells and thus maximise binding of the radioisotope-bearing antibody to CD20+ malignant cells. If required (depending on local regulations), the surrogate complex 111In-ibritumomab tiuxetan (5 mCi [185 MBq]) is infused for gamma imaging to assess biodistribution and for dosimetric study.

Dosimetry and imaging studies using 111In-ibritumomab tiuxetan show generally low uptake of radioactivity by organs throughout the body (in particular, the bone marrow), with rapid appearance and concentration in the tumour. Dosimetry does not correlate with toxicity, and is no longer considered necessary in most centres in the standard use of Y-90 ibritumomab tiuxetan.

The treatment therapeutic component, Y-90 ibritumomab tiuxetan is calculated based on body weight (0.4 or 0.3 mCi/kg [14.8 or 11.1 MBq/kg]; maximum dose 32mCi [1184MBq]) and infused after a ‘cold’ therapeutic dose of rituximab (250 mg/m2). This sub-therapeutic dose of unlabelled rituximab administered (250 mg/m2) is about three quarters of that used when rituximab is given as a treatment for low-grade NHLs (375 mg/m2).

In a Phase I/II dose-escalation trial in 51 patients with low-grade, intermediate-grade, or mantle-cell NHL, Y-90 ibritumomab tiuxetan given at 0.2 to 0.4 mCi/kg (7.4-14.8 MBq/kg) produced an overall response rate of 67% (26% CR), with response durations ranging from 10.8 to 14.4 months. The response rate was highest in patients with low-grade NHL, with an overall response rate of 82% (27% CR, 56% PR) compared with 43% in patients with intermediate-grade NHL (29% CR, 14% PR) [4].

A Phase III study involving 143 patients compared Y-90 ibritumomab tiuxetan with single-agent rituximab in patients with relapsed or refractory low-grade, follicular, or transformed CD20+ NHL. The Y-90 ibritumomab tiuxetan treatment resulted in a response rate of 80% (30% CR) compared with an overall response rate of 56% (16% CR) for rituximab therapy (*p*=.002). The highest response rate was obtained in patients with follicular lymphomas (86% vs. 67% in non-follicular NHL). Rituximab produced a response rate of 55% in patients with follicular lymphomas (*p*<.001 vs. Y-90 ibritumomab tiuxetan) and 50% in non-follicular NHLs [5].

Long-term data have been reported from the Phase I/II dose-escalation trial of Y-90 ibritumomab tiuxetan in 51 patients with low-grade, diffuse large-cell, or mantle-cell NHLs, up to a median follow-up of 28.5 months with up to 63 months for ongoing responders. The response rate was 73%, (51% CR/CRu) in all patients, 85% (58% CR/CRu) in patients with FL, and 58% (50% CR/CRu) in patients with diffuse large cell lymphoma (DLCL). Median time to progression for all patients, treated at the recommended dose of 0.4 mCi/kg (14.8 MBq/kg), was 28.3 months with those ongoing responders having a median time to progression of 45.0 months [6].

### Schedule of I-131 tositumomab therapy and clinical efficacy

The standard schedule for I I-131 tositumomab involves a dosimetric dose of 185 MBq of I-131 tositumomab given with pre-administration of unlabelled antibody, followed by total body imaging for dosimetry calculations. The dosimetric scans are performed over the next few consecutive days. This enables the estimation of the radiopharmaceutical clearance time and the therapeutic dose required to deliver a fixed total body radiation dose (usually 65 or 75 cGy). The therapy dose is administered one week post-dosimetric dose, again with pre-medication with unlabelled antibody. Thyroid blockage with Lugols’ iodine is necessary for at least three weeks from the dosimetric dose. In view of the gamma emissions of I-131, in most centres, the infusion of the therapeutic dose is conducted with hospital isolation of the patient until the radiation exposure has dropped to acceptable levels.

A Phase I/II single centre study trial of I-131 tositumomab found that chemotherapy-relapsed or chemotherapy-refractory low-grade or transformed low grade NHLs with a median of 4 prior to chemotherapy regimes had a response rate of 83% and a complete response rate of 48%. The median progression-free survival was 14 months for responders and 20 months for complete responders. Seven of 20 complete responders continued CR for 3 to 5.7 years [7-9].

A Phase II multi-centre study also showed an overall response rate of 57% and a complete response rate of 32%.10 A Phase III clinical trial of Bexxar in chemotherapy-refractory low-grade or transformed low grade B-cell NHLs gave a response rate of 65% and a complete response rate of 20% compared to 28% response rate and 3% complete response rate in the patients’ last qualifying chemotherapy regimens [11].

### Other RIT for haematological indications

Apart from the anti-CD20 monoclonal antibody, the anti-CD22 antibody (epratuzumab) with various radiolabels including Y-90, has also been used in RIT in non-Hodgkin’s lymphoma with promising results [12].

In therapeutic options for acute leukaemia, research on RIT largely focussed on the myeloid antigen CD33. Monoclonal antibodies M195 and HuM195 raised against this target, radio-labelled with I-131, have been used in the conditioning regime for allogenic bone marrow transplantation together with busulfan and cyclophosphamide. There was good targeting of radiotracer to the marrow, liver and spleen with absorbed marrow doses of 272-1470 cGy [13].

Another group working with anti-CD66 RIT recently published a Phase I/II study where Rhenium-188 or Y-90 labelled anti-CD66 antibody was used a part of a dose-reduced conditioning regimen for patients with acute leukaemia or myelodysplastic syndrome. The authors concluded that RIT using the anti-CD66 antibody was feasible and safe in their elderly patient group and provided a high marrow dose [14].

### RIT for solid tumours

The results of RIT for several solid tumour types have also been published. The tumour types included ovarian, colorectal and glioma tumours [15-17]. Currently, there is no sufficient evidence to suggest good clinical activity for advanced metastatic disease of these tumour types, although the potential for future development of these agents for early or adjuvant therapy for micro-metastases remains bright. Solid organ RIT may become useful in an adjuvant setting, where primary surgical resection of the tumour has been successfully performed.

## FUTURE DIRECTIONS

RIT is a promising new radioisotope oncological therapy. New ways to improve the efficacy of these treatments should be sought. One of the ways in which this can be achieved is through the use of combination therapy, i.e., adding chemotherapy to RIT, perhaps as frontline or in salvage chemotherapeutic regimes. An attractive combination, particularly for clearing circulating cells or bone marrow cells in preparation for the administration of the radiation, is the addition of a radiosensitiser, such as fludarabine or paclitaxel. In three different mouse models (lymphoma, breast, prostate) paclitaxel has been shown to enhance the effects of RIT [18]. Athymic mice bearing Raji xenografts were treated with Y-90-Lym-1 alone; paclitaxel alone; Y-90-Lym-1 plus paclitaxel; or given no treatment. The addition of the radiosensitiser to RIT markedly improved survival compared with either treatment alone. Survival was 71% for Y-90-Lym-1 plus paclitaxel; 29% for paclitaxel alone; 6% for Y-90-Lym-1 alone; and 14% in the untreated group. The average tumour volume in the RIT plus radiosensitiser group was reduced by 89% compared with RIT alone; and by 99% compared with radiosensitiser alone [19].

The role of RIT in the overall sequence of therapeutic interventions and clinical treatment protocols for non-Hodgkin’s lymphomas (such as in the neo-adjuvant setting or adjuvant setting) should be examined in the context of randomised prospective trials. New work on molecular and cellular biology will provide further impetus to the development of new areas of RIT in the future.

Another recent development is the use of α-emitting radionuclides, such as bismuth-212 and bismuth-213 in RIT [20]. They are used because of the highly ionising nature of their particulate radiation. (α-radiation are relatively heavier helium nuclei, compared to β-radiation which are electrons). These heavier nuclei transfer larger amounts of energy per unit path-length in tissues and are stopped much earlier by tissue than electrons. Based on these considerations, α-isotopes would be highly relevant when targeting dispersed but small volume tumours.

## CONCLUSION

RIT is a highly promising oncological therapeutic modality, with good reported efficacy for refractory or relapsed NHLs. Future randomised, prospective trials involving RIT will further determine and almost certainly expand the scope of this useful therapeutic modality in the clinical management of NHL and other tumours.
